# Cis and Trans Regulatory Mechanisms Control AP2-Mediated B Cell Receptor Endocytosis via Select Tyrosine-Based Motifs

**DOI:** 10.1371/journal.pone.0054938

**Published:** 2013-01-23

**Authors:** Kathleen Busman-Sahay, Lisa Drake, Anand Sitaram, Michael Marks, James R. Drake

**Affiliations:** 1 Center for Immunology and Microbial Disease, Albany Medical College, Albany, New York, United States of America; 2 Departments of Pathology and Laboratory Medicine and Physiology, University of Pennsylvania School of Medicine, Philadelphia, Pennsylvania, United States of America; Thomas Jefferson University, United States of America

## Abstract

Following antigen recognition, B cell receptor (BCR)-mediated endocytosis is the first step of antigen processing and presentation to CD4+ T cells, a crucial component of the initiation and control of the humoral immune response. Despite this, the molecular mechanism of BCR internalization is poorly understood. Recently, studies of activated B cell-like diffuse large B cell lymphoma (ABC DLBCL) have shown that mutations within the BCR subunit CD79b leads to increased BCR surface expression, suggesting that CD79b may control BCR internalization. Adaptor protein 2 (AP2) is the major mediator of receptor endocytosis via clathrin-coated pits. The BCR contains five putative AP2-binding YxxØ motifs, including four that are present within two immunoreceptor tyrosine-based activation motifs (ITAMs). Using a combination of *in vitro* and *in situ* approaches, we establish that the sole mediator of AP2-dependent BCR internalization is the membrane proximal ITAM YxxØ motif in CD79b, which is a major target of mutation in ABC DLBCL. In addition, we establish that BCR internalization can be regulated at a minimum of two different levels: regulation of YxxØ AP2 binding *in cis* by downstream ITAM-embedded DCSM and QTAT regulatory elements and regulation *in trans* by the partner cytoplasmic domain of the CD79 heterodimer. Beyond establishing the basic rules governing BCR internalization, these results illustrate an underappreciated role for ITAM residues in controlling clathrin-dependent endocytosis and highlight the complex mechanisms that control the activity of AP2 binding motifs in this receptor system.

## Introduction

B lymphocytes are professional antigen presenting cells that present peptide–MHC class II complexes to CD4^+^ T cells to drive antibody class switching and affinity maturation. Efficient B cell antigen presentation depends upon initial BCR-mediated antigen binding and internalization [1]–[3]. The BCR is composed of an antigen binding subunit made up of a membrane-bound immunoglobulin molecule, which is non-covalently associated with the CD79a/CD79b heterodimer that drives BCR signaling [4]. Naïve B cells co-express an IgM and IgD BCR. The cytoplasmic domains of the antigen binding subunit of these BCR molecules are short (*i.e.*, lysine-valine-lysine, KVK). Therefore, antigen-induced BCR signaling and endocytosis are mediated through the cytoplasmic domains of CD79. Antigen binding results in clustering of plasma membrane BCR molecules, driving CD79 ITAM tyrosine phosphorylation by Src family kinases such as Lyn [5], recruitment of Syk [6] and initiation of multiple downstream signaling pathways. However, cross-linking results in ITAM phosphorylation of only a small fraction of antigen-engaged BCR molecules, which are then retained at the cell surface. In contrast, a large fraction of antigen-BCR complexes remain unphosphorylated and are rapidly cleared from the cell surface [7].

Concomitant with BCR signaling, antigen-BCR (Ag-BCR) complexes are also internalized, which is the first step in antigen delivery to MHC class II-containing compartments for conversion to peptide-MHC class II complexes. However, the mechanism of BCR internalization is only poorly understood. BCR endocytosis appears to be primarily driven by clathrin-coated pits (CCP) [8]–[10], though a clathrin-independent pathway may be utilized to a lesser extent [11], [12]. While BCR signaling and endocytosis occur concurrently, the relationship between the two processes is controversial. This lab and others have shown that the majority of BCR endocytosis occurs independent of BCR signaling [7], [9], [10]. However, other studies have suggested that BCR endocytosis requires signaling [13], specifically for the recruitment of proteins such as actin [14], Vav and dynamin [15], or possibly to drive clathrin phosphorylation [16], [17]. Thus, while clathrin coated pits appear to represent the primary pathway for BCR endocytosis, the mechanism by which Ag-BCR complexes access CCP and how this is regulated remains unclear.

Clathrin-coated pits are an assembly of clathrin ‘triskelions’, which are composed of three clathrin heavy chains and three clathrin light chains [18]. Triskelions assemble into a polyhedral lattice that stabilizes vesicle formation. However, clathrin does not directly bind endocytic receptors. Instead, endocytic cargo is recognized by adaptor proteins such as AP2 [19], which interact with the receptor and recruit clathrin triskelions to the forming CCP [20]. The predominant endocytic clathrin adaptor is AP2, which interacts with one of two common motifs in the cytoplasmic domain of a receptor, an acidic dileucine motif conforming to the consensus E/DxxxLL/I or a YxxØ motif where Ø represents a bulky, hydrophobic amino acid such as leucine or isoleucine. Each AP2 complex is composed of two large (α and β), one medium (µ), and one small (σ) subunit [21]. Moreover, the solved crystal structures of AP2 bound to various endocytosis motifs clearly distinguish the YxxØ binding site on the µ subunit [22] from the dileucine-binding site that is largely located on the σ subunit [23]. However, despite several studies into the mechanism of BCR internalization, the adaptor that mediates BCR internalization and the precise location of the adaptor-binding site within the BCR remains unknown.

There are six tyrosines within the cytoplasmic domain of the CD79 heterodimer, five of which lie within a YxxØ putative AP2 binding motif. Each subunit of the heterodimer possesses an ITAM with the consensus sequence [D/E]x_7_[D/E]x_2_YxxLx_7–10_YxxL/I, meaning that each BCR ITAM contains two YxxØ motifs. The remaining YxxØ motif is present downstream of the CD79a ITAM. Both CD79a and CD79b could therefore bind AP2 and mediate BCR internalization. However, the relative contribution of each CD79 subunit to BCR internalization is currently unclear.

The cytoplasmic domain of CD79a clearly contains a functional endocytosis motif, as it can mediate internalization when expressed in isolation [24], [25] or as an artificially constructed homodimer [24], [26]. Moreover, the membrane proximal ITAM tyrosine (*i.e.*, Y182) and the downstream non-ITAM tyrosine (*i.e.*, Y204) of CD79a have been implicated in constitutive [27] and cross-linking-induced [7] BCR endocytosis, respectively. However, *in vivo* analysis of BCR internalization clearly demonstrates a role for the CD79b ITAM tyrosines in both constitutive and inducible BCR endocytosis [28]. Moreover, while CD79b is not internalization competent as a monomeric construct, artificial pairing of two CD79b cytoplasmic domains [24], [29]–[31] or generation of select mutations within CD79b [24] leads to receptor endocytosis with kinetics remarkably similar to wild-type BCR. Finally, a recent study of B cell lymphoma suggests a role for the membrane proximal ITAM tyrosine in CD79b, Y195, in influencing BCR surface expression levels [32]. Thus, there is currently no consensus concerning the molecular mechanism of antigen-induced BCR internalization. While some disparities may be due to variations in the experimental systems, it also suggests that the mechanism of BCR internalization is significantly more complex than originally anticipated.

In the current study, we build upon the recent findings in B cell lymphomas that mutation of a single BCR CD79 tyrosine appears to block BCR internalization [32], and establish the mechanistic details of antigen-induced AP2-mediated BCR internalization. Using multiple *in vitro* and *in situ* approaches, we identify the AP2 binding site of the BCR and distinct mechanisms that control BCR internalization, including regulation *in cis* and *in trans*. These results establish a role for select ITAM tyrosines in cross-linking-induced, clathrin-dependent BCR endocytosis, resolve some of the apparent discrepancies on the mechanism of BCR internalization in the existing literature and establish and highlight the sophisticated mechanisms that control BCR-mediated antigen internalization.

## Materials and Methods

### Ethics Statement

Mice were housed and used in strict accordance to the guidelines established by the Albany Medical College Institutional Animal Care and Use Committee. Animal protocols were reviewed and approved by the Albany Medical College Institutional Animal Care and Use Committee (Protocol #704326).

### Cells for Analysis

A20µWT and IIA1.6 cells were grown and used as previously reported [33], [34]. Splenocytes from B10.Br mice (purchased from Jackson Laboratories and housed in the AMC Animal Resources Facility) were harvested and used as previously reported [34].

### Electron Microscopy

EM analysis of AP2-BCR association within CCP was performed via a modification of a previously reported approach [10]. In brief, A20µWT cells or B10.Br-derived splenocytes were stimulated with rabbit anti-human IgM-btn (309-065-095; Jackson ImmunoResearch Laboratories, Inc., West Grove, PA, USA) or mouse anti-mouse IgM^b^-btn (AF6-78; BD Pharmingen, San Jose, CA, USA), respectively, plus goat anti-btn-15 nm gold (25253; Electron Microscopy Services [EMS], Hatfield, PA, USA) for 5 minutes at 37^o^C, then cooled. The stained cells were then pressed between alcian blue-coated coverslips and lysine-coated nickel EM grids (FCF300-Ni-50; EMS) and quickly separated. The plasma membrane remaining on the copper grids was fixed and stained with 1% solutions of OsO4, tannic acid and uranyl acetate. AP2 was detected by staining the exposed cytosolic surface of the membrane using mouse anti-mouse α-adaptin (AP.6; Abcam, Cambridge, MA, USA) and Protein A-5 nm gold (courtesy of Dr. Paul Webster). CCP were distinguished by morphology and gold particles within CCP were enumerated manually on negatives from images acquired using a JEOL 100CXII STEM microscope.

### Plasmid Generation and Mutagenesis

Yeast two-hybrid: The cytoplasmic domain of murine CD79a or CD79b was PCR amplified and separately inserted into the multiple cloning site (MCS) of the pGBT9 yeast two-hybrid plasmid, which contains the Gal4 DNA binding domain and a tryptophan marker. Murine AP2µ was PCR amplified and inserted into the MCS of the pACT2 plasmid, which contains the Gal4 activation domain and a leucine marker. GST-CD79: The cytoplasmic domains of CD79a, CD79b, or portions thereof, were PCR amplified and separately inserted into the multiple cloning site downstream of GST and the Factor Xa cleavage sequence in the pGEX-5x vector (GE Healthcare Life Sciences). MHC class II-CD79 chimera: The MHC class II-CD79 constructs were a gift from Dr. John Cambier and modified to express the immunodominant epitope from hen egg lysozyme (HEL), residues 47–62, and a poly-glycine linker at the N-terminus of MHC class II β chain. This was done to prevent association of the class II α/β heterodimer with invariant chain, which also contains internalization/AP2-binding motifs. The sequence of the HEL tether in the context of WT MHC class II and the cytoplasmic deletion version of the MHC class II were previously reported [33]. Site directed mutagenesis of the GST-CD79 and MHC class II-CD79 constructs was performed with the Stratagene QuickChange kit (Agilent Technologies, Santa Clara, CA, USA) according to the manufacturer’s instructions.

### Yeast Two-hybrid

The *Saccharomyces cerevisiae* strain HF7c (Clontech, Mountain View, CA, USA) was maintained on complete YPD plates. Co-transformation with pGBT9-CD79 and pACT2-AP2µ plasmids was performed by a modification of the lithium acetate procedure as described in the Yeast Protocols Handbook from Clontech. HF7c transformants were selected by spreading on plates lacking leucine and tryptophan. For colony growth assays, HF7c transformants were pooled and spotted once or in 5-fold serial dilutions on plates lacking leucine, tryptophan, and histidine, and allowed to grow at 30°C for 3–5 days.

### AP2 Binding Assay

Biotinylated AP2µ subunit was generated using the TnT Transcription/Translation System combined with the Transcend Biotinylated tRNA (Promega, Madison, WI, USA). *E. coli* BL21 (Invitrogen, Grand Island, NY, USA) expressing the GST-fusion proteins were grown and induced with IPTG according to the manufacturer’s protocol. The bacteria were lysed by sonication in a buffer containing 0.5% NP-40, 20mM Tris pH 8.0, 100mM NaCl and 1mM EDTA. GST-fusion proteins were bound to glutathione beads, washed and incubated with biotinylated AP2µ (AP2µ-btn) for 30 minutes. Following washing, the beads were boiled in reducing SDS-PAGE sample buffer and the eluted proteins run on SDS-PAGE and transferred to nitrocellulose membranes. Ponceau S was used to detect total GST protein loading and AP2µ-btn was detected by western blot with streptavidin-HRP and ECL (Pierce, Thermo Scientific, Rockford, IL, USA). For the competitive inhibition assay, peptides were synthesized by Anaspec (Fremont, CA, USA) and, following the protocol of [35], resuspended to 1 mM in 0.05% Triton-X-100, 50 mM HEPES (pH 7.3), 10% glycerol, 100 mM KCl, 2 mM MgCl_2_, 0.1 mM CaCl_2_ at pH 7.3. The peptides were mixed with AP2µ-btn for 30 minutes and this mixture was then added to the washed glutathione-immobilized GST-fusion complex for 30 minutes, followed by the remainder of the basic protocol outlined above.

### Confocal Microscopy and Image Analysis

IIA1.6 cells, an FcR^–^ derivative of the A20 murine B cell lymphoma line, were transiently transfected via electroporation with 10 µg of each of the isolated MHC class II–CD79a and -CD79b chain constructs, or mutants thereof. After 20–24 hours, the cells were collected, washed, and incubated with mouse anti-mouse I-A^k^–Alexa Fluor 488 (10-3.6; BioLegend, San Diego, CA, USA) for 15 minutes at 37^o^C. The cells were cooled on ice (and remained chilled for the remainder of the staining protocol), washed and stained with anti-mouse IgG_2a_-Alexa Fluor 594 (Invitrogen), which will stain both primary antibody on the cell surface and the endogenous BCR. The cells were adhered to Alcian Blue-coated coverslips, fixed and mounted onto microscope slides with Fluoro-gel mounting media (EMS). Images are Z stacks of 1.5 µm slices obtained on an Olympus Fluoview (Center Valley, PA, USA) FV1000 with a 60x N.A. 1.23 water immersion lens at room temperature. Individual cells were photographed using an 8x optical zoom and the presented images depict an internal section with the highest Alexa Fluor 488-only staining. Three independent experiments with a total of 100+ cells were imaged, and only transfected cells containing equal or greater internal staining compared to external staining were marked as containing internalized receptor.

### Flow Cytometry

Internalization was monitored as previously reported [33]. Briefly, chimeric surface MHC class II-CD79 molecules of transiently transfected cells were tagged with 10-3.6-btn, the cells incubated at 37°C for the indicated time, and then non-internalized 10-3.6-btn detected with SA-PE. Reported is the decrease in MFI of SA-PE staining normalized to time 0.

### Statistics

Student’s *t* test was used to measure statistical significance using Microsoft Excel 2008 for Mac v12.3.3. Statistical significance is designated as * p≤0.05, ** p≤0.01, and *** p≤0.001.

## Results

### CD79b Y195 Drives BCR Internalization

**Figure 1:**
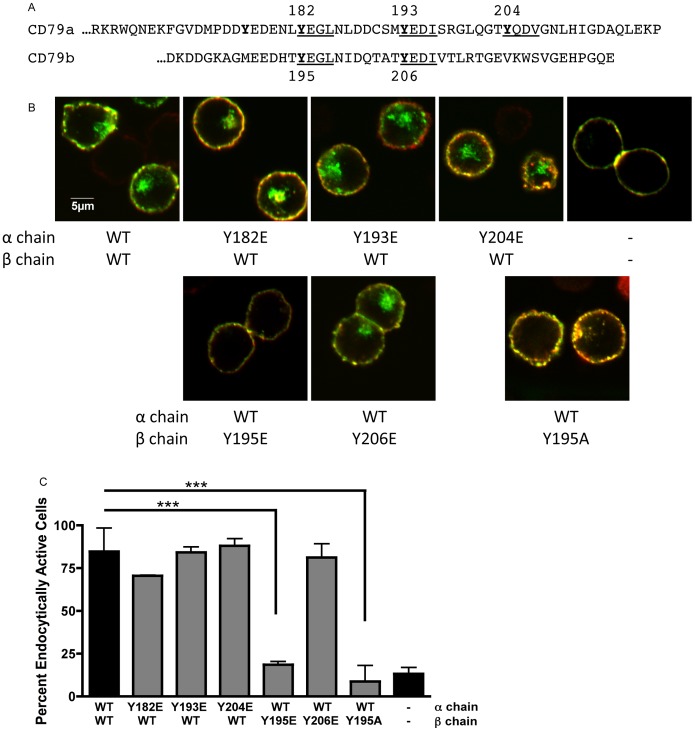
CD79b Y195 Controls BCR Internalization. Panel A, Amino acid sequences of the cytoplasmic domains of CD79a and CD79b. YxxØ putative AP2 binding motifs underlined. Panel B, Confocal laser scanning microscopic analysis of the endocytosis of indicated MHC class II-CD79 chimeric proteins. Plasma membrane is yellow (anti-MHC class II-Alexa 488+ post-endocytic labeling of external MHC class II-CD79 with an Alexa 594-labeled antibody). Internalized MHC class II-CD79 is green (anti-MHC class II-Alexa 488 only). Panel C, Quantification of MHC class II-CD79 endocytosis. 100+ cells from across 3 independent experiments were scored for internalization. Reported is the percent of cells showing internalized MHC class II-CD79 for each construct. Statistical comparisons were made between the construct with both CD79 cytoplasmic domains and cells expressing other constructs.

The cytoplasmic domains of the IgM and IgD BCR expressed by naïve B cells consist of four polypeptide chains; two immunoglobulin heavy chains (which have short cytoplasmic domains of the sequence KVK) and those of CD79a and CD79b (which are ∼61 and 48 amino acids in length, respectively; Figure 1A). The cytoplasmic domains of CD79a and CD79b contain the two BCR ITAMs that include four of the receptor’s five YxxØ putative endocytosis motifs.

A recent publication reported that activated B cell-like diffuse large B cell lymphomas (ABC DLBCL) exhibit increased BCR surface expression, suggesting a defect in constitutive (and possibly antigen-driven) BCR internalization. The same publication also reported that a large portion of ABC DLBCL patients have a point mutation in the membrane proximal ITAM tyrosine of CD79b (CD79b Y195, which is part of a YxxØ putative endocytosis motif) but not of the corresponding membrane-proximal ITAM tyrosine of CD79a. To determine if there is a link between CD79b Y195 and antigen-driven BCR internalization, the impact of mutating each CD79 membrane proximal ITAM tyrosine (*i.e.* CD79a Y182 or CD79b Y195) on cross-linking-induced BCR internalization was directly tested. We used a model system in which the cytoplasmic domains of CD79a and CD79b are grafted onto the extracellular and transmembrane domains of an MHC class II molecule (Figures 1B and 1C and Figure S1). Here, it should be noted that class II molecules form only α/β heterodimers. Thus, homodimeric reporter molecules bearing either two CD79a or two CD79b cytoplasmic domains will *not* form under these conditions. Consistent with the notion that the membrane-proximal ITAM tyrosine of CD79b might drive BCR internalization, mutation of CD79b Y195 to either glutamic acid (E, a mutation very similar to the tyrosine to aspartic acid [D] mutation found in multiple ABC DLBCLs) or to alanine (A, a more conservative substitution) completely blocks CD79-driven internalization. Conversely, mutation of the homologous tyrosine in CD79a (*i.e.*, Y182) to glutamic acid has no effect on BCR internalization. Moreover, individual mutation of any of the other receptor YxxØ tyrosine residues to glutamic acid also has no impact on receptor internalization. When combined with the reported increase in BCR surface expression in ABC DLBCLs bearing similar point mutations, these results suggest that CD79b Y195 is a critical element of the sole active endocytosis motif of the BCR, whereas CD79a Y182 plays little direct role in the endocytosis of the CD79a/b heterodimer.

### AP2 Mediates BCR Internalization

**Figure 2:**
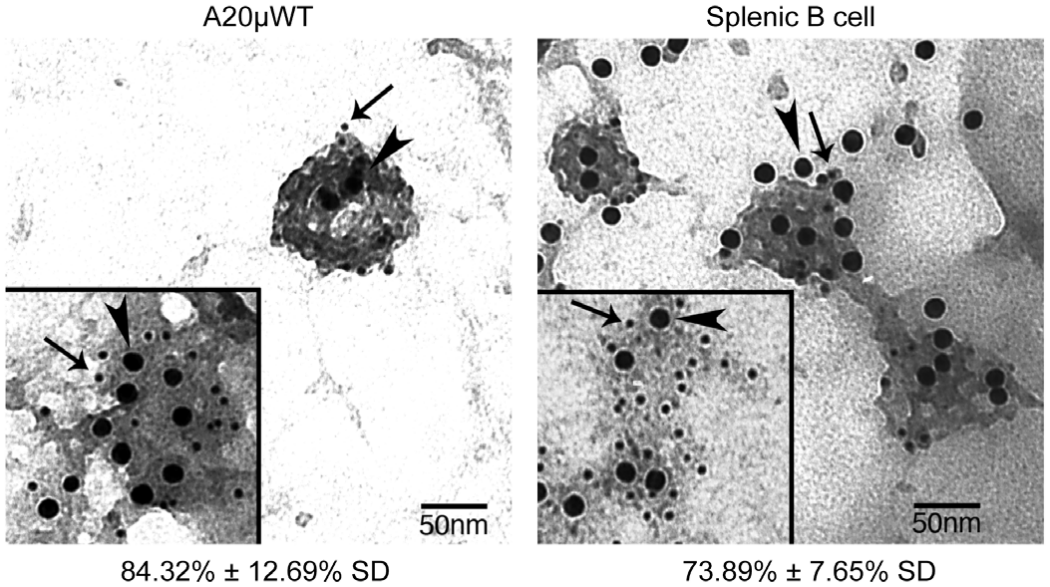
Ultrastructural Colocalization of Ligand-BCR Complexes and AP2 in Clathrin-Coated Pits. A20µWT B cells or primary murine splenocytes were pulsed with anti-IgM-btn followed by anti-biotin-15 nm gold (arrow heads), incubated 2 minutes at 37° and then plasma membrane rips were prepared as previously reported [10]. The exposed cytoplasmic face of the plasma membrane was stained with anti-AP2 and Protein A-5 nm gold (arrows). The percent of BCR-containing CCP that also stained for AP2 is indicated below each image. Inset: AP2 and BCR co-localization within electron dense membrane regions lacking discernable CCP architecture. Shown are representative images from 1 of 3 experiments, with 1,000+ BCR-bound gold particles or 100+ CCP photographed cumulatively.

The finding that mutation of the membrane-proximal ITAM tyrosine of CD79b, which falls within a putative AP2-binding YxxØ endocytosis motif, blocks all BCR internalization suggests that AP2 is the endocytic adaptor protein mediating BCR internalization. To determine if this is the case, we first used electron microscopy to determine if ligand-BCR complexes and the AP2 adaptor colocalize to plasma membrane CCP (Figure 2). In the A20µWT B cell line that bears a phosphorylcholine-specific human IgM BCR, over 80% of CCP that contain antigen-BCR complexes also stain for AP2. In normal splenic B cells, the level of BCR–AP2 colocalization in CCP is almost 75%. We also find significant BCR–AP2 colocalization outside of morphologically-identifiable CCP (Figure 2 insets), suggesting that in B cells, like other cells, receptors such as the BCR may interact with patches of membrane-associated AP2 and stabilize them, allowing subsequent clathrin recruitment and maturation into a CCP. Together with the finding that mutation of a putative AP2 binding motif (*i.e.* CD79b Y195) can completely block BCR internalization, these results suggest that the YxxØ motif centered on the membrane proximal ITAM tyrosine of CD79b binds AP2 to support CCP-mediated BCR internalization.

### CD79a/b Pairing Impacts AP2 Binding and Endocytosis

**Figure 3:**
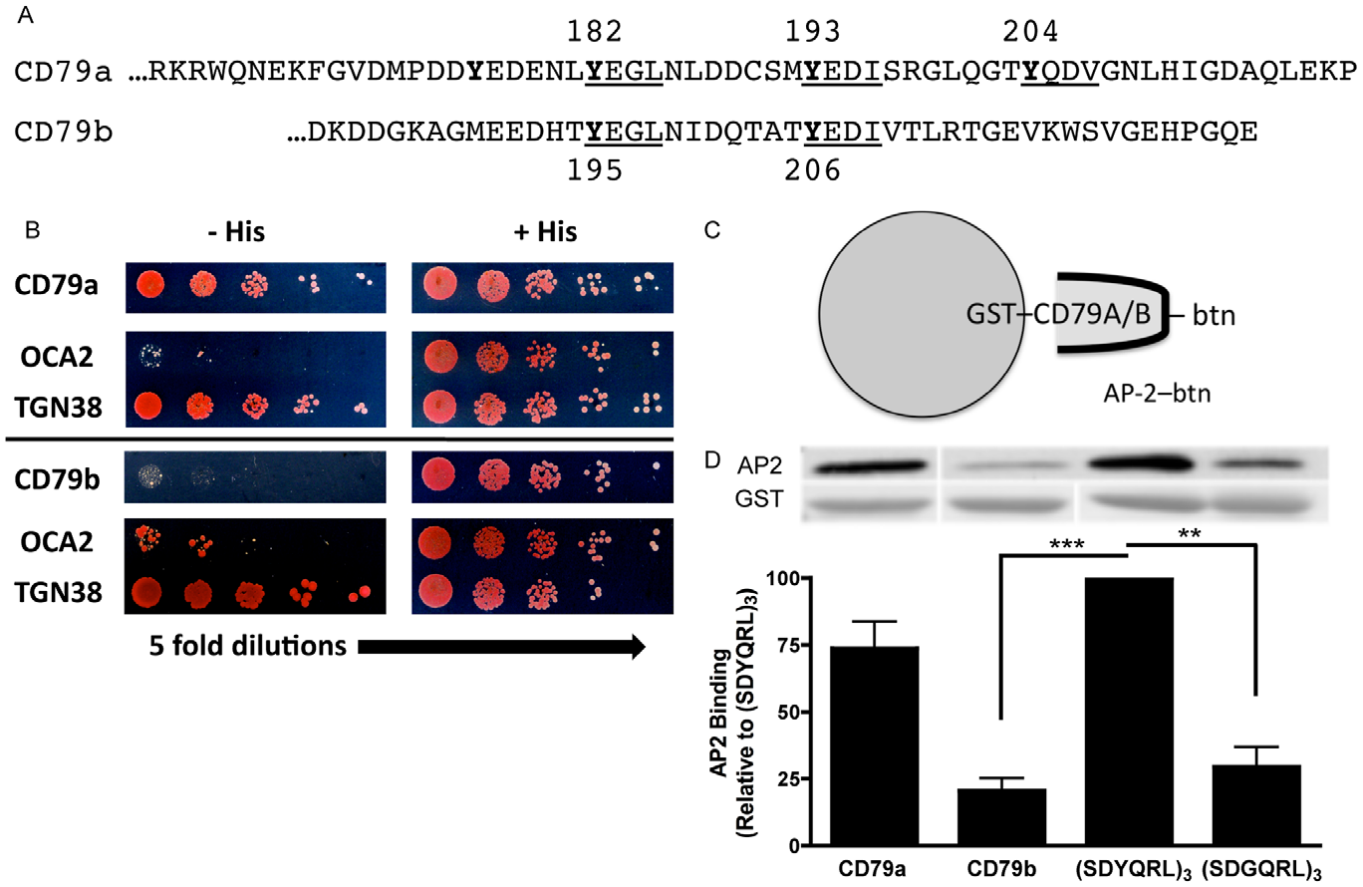
AP2µ Binds to Isolated CD79a but not CD79b. Panel A, Amino acid sequences of CD79a and CD79b cytoplasmic domains. YxxØ putative AP2 binding motifs underlined. Panel B, AP2µ expressed as a Gal4 activation domain fusion protein was assayed for specific interaction with the cytoplasmic domain of either CD79a or CD79b fused to the Gal4 DNA binding domain. Growth on histidine deficient (His-) plates indicates an AP2–CD79 interaction. The cytoplasmic domain of TGN38 contains a known AP2 binding YxxØ motif and served as a positive control, while the cytoplasmic domain of OCA2 contains a dileucine motif (which does not bind AP2µ) and served as a negative control. Data are representative of 2 experiments. Panel C, Diagram of the GST-CD79 cytoplasmic domain–AP2µ direct binding assay. Panel D, The cytoplasmic domains of CD79a and CD79b were expressed as GST fusion proteins in BL21 *E. coli* cells. GST-fusion proteins were captured from cell lysates on glutathione beads and the resulting matrix was tested for binding to *in vitro* translated, biotin-labeled AP2µ. The AP2 binding motif from TGN38, (SDYQRL)_3_, and a non-AP2-binding derivation containing a tyrosine to glycine substitution, (SDGQRL)_3_, fused to GST served as positive and negative controls, respectively. Binding is expressed as a percentage of (SDYQRL)_3_–AP2 interactions. Data is the mean of 3 independent experiments ± S.E.M. Statistical comparisons were measured between SDYQRL and other samples.

The combined cytoplasmic domains of CD79a and CD79b possess a total of five YxxØ motifs that may bind AP2 (Figure 3A), yet the membrane proximal ITAM tyrosine of CD79b (*i.e.*, Y195) appears to be the only motif that drives AP2-mediated BCR internalization. To confirm the role of the AP2 adaptor in BCR internalization and more precisely map the site of AP2 binding, the interaction of AP2 with the individual cytoplasmic domains of CD79a and CD79b was investigated. The first step was to use a yeast two-hybrid assay to determine whether the cytoplasmic domain of CD79a and/or CD79b interacts directly with the µ subunit of AP2 (AP2µ, the AP2 subunit known to bind YxxØ motifs; Figure 3B). Surprisingly, when tested in isolation the cytoplasmic domain of CD79b fails to interact with AP2µ, whereas the cytoplasmic domain of CD79a exhibits strong AP2µ binding. These results were unexpected based on the observation that mutation of the membrane proximal ITAM tyrosine of CD79b blocks BCR internalization (Figure 1). To confirm the yeast two-hybrid results and rule out the possibility that the unexpected pattern of AP2 binding might be due to the nuclear environment where the interaction took place, an *in vitro* AP2 binding assay was used to further address the question. As diagrammed in Figure 3C, the cytoplasmic domain of either CD79a or CD79b was linked to GST and the resulting fusion protein was captured on glutathione beads. The ability of biotin-labeled AP2µ (AP2µ-btn) to bind these CD79-coated beads was then determined (Figure 3D). Consistent with the yeast two-hybrid results, the *in vitro* AP2 binding assay confirmed the ability of the cytoplasmic domain of CD79a, but not that of CD79b, to directly bind AP2.

**Figure 4:**
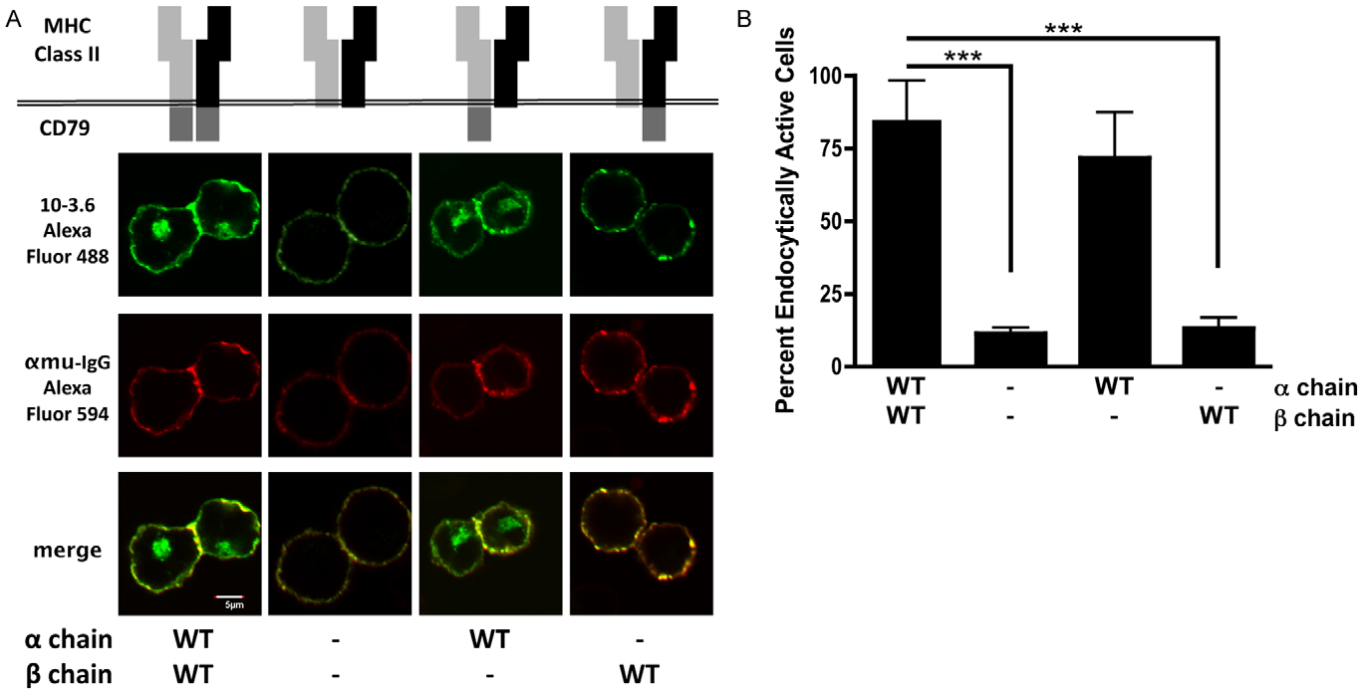
Receptor Endocytosis of Isolated CD79a but not CD79b. Endocytosis of the indicated MHC class II-CD79 fusion proteins was analyzed (Panel A) and quantitated (Panel B) as in Figure 1. Statistical comparisons were made between the reporter proteins with both CD79 cytoplasmic domains and other reporter proteins.

These results were extended to the *in situ* condition by grafting the cytoplasmic domain of either CD79a or CD79b onto an MHC class II molecule (which does *not* form α-α or β-β chain homodimers) and determining the internalization capacity of the chimeric molecules (Figures 4A and 4B). When tested in isolation, the cytoplasmic domain of CD79a, but *not* that of CD79b, is able to mediate cross-linking-induced receptor internalization. Taken together with the results presented in Figure 1, which establish that mutation of CD79b Y195 to either glutamic acid or alanine blocks BCR internalization, the results presented in Figures 3 and 4 suggest that in the context of the intact CD79 heterodimer the presence of CD79a is required to “activate” the AP2 binding properties of CD79b (*i.e.*, that CD79a positively regulates the AP2 binding properties of CD79b *in trans*). The alternative, more convoluted, scenario is that endocytosis of the CD79 heterodimer is driven by AP2 binding to a YxxØ within CD79a and that this AP2-CD79a interaction is inhibited by mutation of CD79b Y195 to glutamic acid. However, this possibility is ruled out both by the observation that a more conservative CD79b Y195A mutation also blocks heterodimer internalization and by the finding that mutation of any individual CD79a tyrosine to glutamic acid fails to block endocytosis of the CD79 heterodimer (Figure 1). These results establish the central role of AP2 in mediating BCR internalization and also reveal that the endocytic activity of each CD79 cytoplasmic domain can be controlled *in trans* by the presence or absence of the partner cytoplasmic domain. Furthermore, this finding suggests that there is a complex interaction between the two CD79 cytoplasmic domains that controls the endocytic activity of the various BCR AP2 binding motifs.

### Flanking Regulatory Elements Control BCR Endocytosis Motif Activity

**Figure 5:**
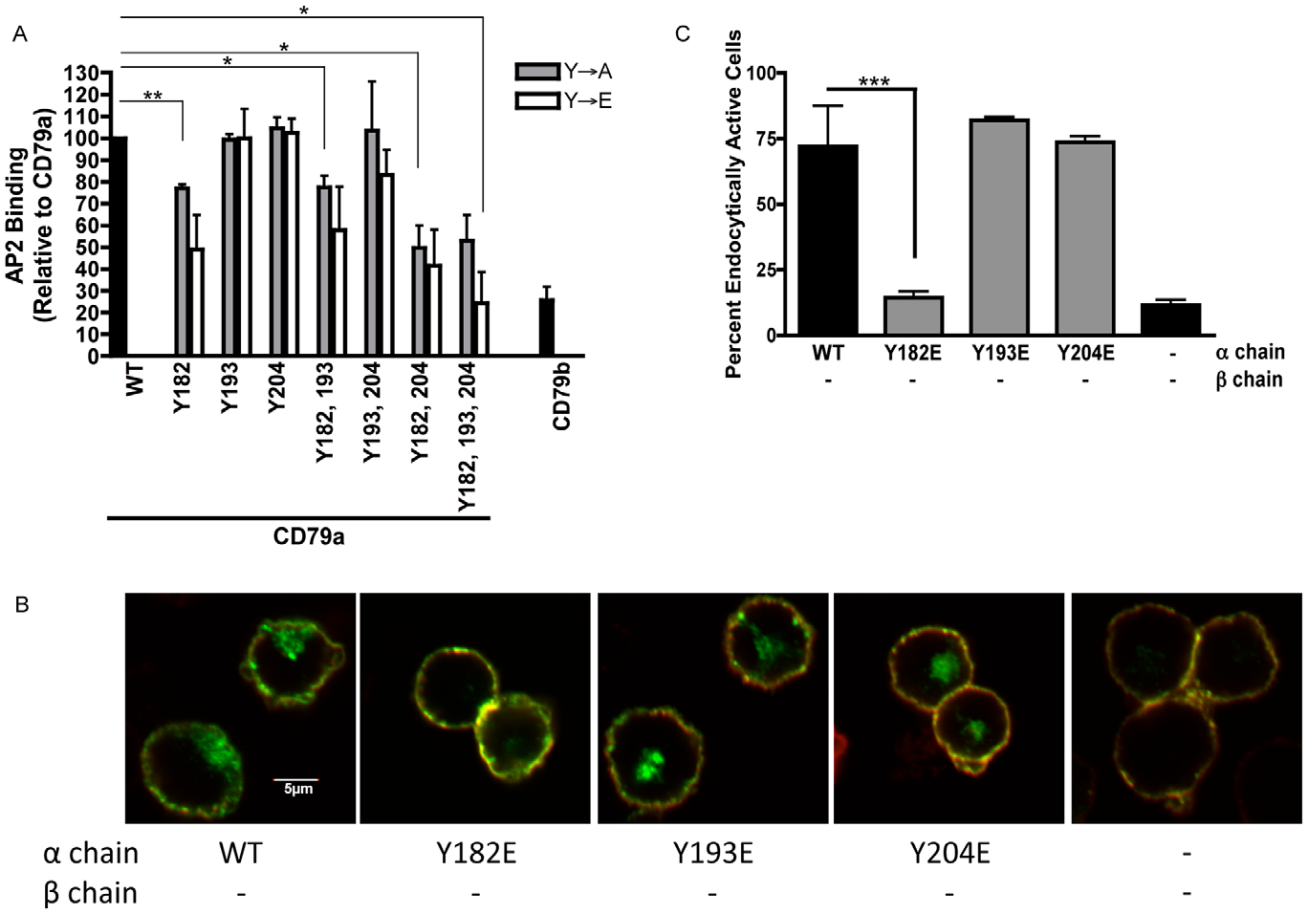
The Membrane-Proximal YxxØ Motif of Isolated CD79a Mediates AP2 Binding and Endocytosis. Panel A, The binding of AP2µ-btn to bead-captured GST bearing full length CD79a with the indicated mutations was determined as in Figure 3. Panels B and C, Endocytosis of the indicated MHC class II-CD79 fusion proteins was analyzed and quantitated as in Figure 1. Statistical comparisons were made between the reporter protein expressing the wild type CD79a cytoplasmic domains and other reporter proteins.

Given the surprising finding that CD79a, not CD79b, binds AP2 and is endocytically active when expressed in isolation (Figures 3 and 4), the mechanism of “activation” of the isolated CD79a cytoplasmic domain was investigated. This line of investigation should also allow us to better understand the potential mechanism of “inactivation” of the isolated CD79b cytoplasmic domain as well as regulation of the endocytic activity of the complete BCR. The first step of this process was to identify the YxxØ motif driving CD79a-mediated internalization. To this end, the impact of tyrosine mutation of the three YxxØ motifs in the cytoplasmic domain of CD79a on *in vitro* AP2 binding was determined. Similar to the finding that the membrane-proximal ITAM tyrosine of CD79b is the sole driver of endocytosis in the context of the CD79 heterodimer, the membrane proximal ITAM tyrosine of CD79a (*i.e.*, Y182) is central to the AP2 binding capacity of the isolated CD79a cytoplasmic domain (Figure 5A). While combined mutation of CD79a Y182 and Y204 may have a slightly greater negative impact on AP2 binding than mutation of CD79a Y182 alone, it appears that the majority of AP2 binding is driven by the membrane proximal ITAM tyrosine of CD79a. This analysis was extended to the *in situ* condition by determining the impact of a similar set of mutations on the endocytosis of the MHC class II reporter protein bearing a single isolated CD79a cytoplasmic domain (Figures 5B and 5C). The findings from this *in situ* analysis are fully consistent with those of the *in vitro* AP2 binding assay and establish that the membrane proximal YxxØ motif of CD79a is central to its endocytic activity when expressed in the absence of CD79b.

**Figure 6:**
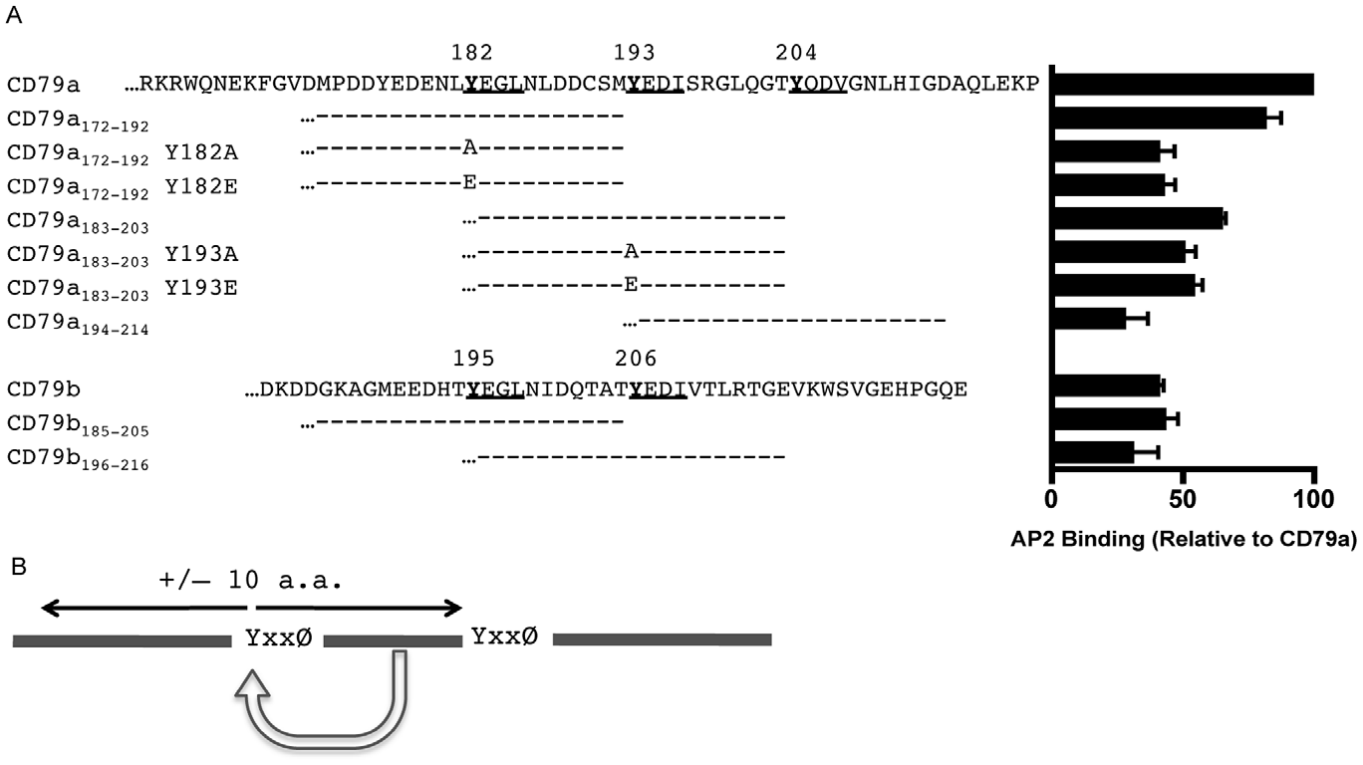
AP2µ Directly Binds the YxxØ Motif Centered on the Membrane-Proximal Tyrosine of Isolated CD79a. Panel A, The binding of AP2µ-btn to bead-captured GST bearing 21 amino acid peptides centered on the five CD79 YxxØ motifs was determined as in Figure 3. Binding is expressed as a percentage of CD79a–AP2 interactions and represents the mean of 3 independent experiments ± SEM. Statistical comparisons were measured between CD79a and other samples. Panel B, For both CD79a and CD79b, a positive or negative regulatory motif lies within +/−10 amino acids of the tyrosine residue of the membrane-proximal YxxØ AP2 binding motif. In this example, the motif is arbitrarily depicted as being downstream of the YxxØ motif.

The results on CD79a-driven endocytosis presented in Figure 5 could either mean that mutation of the membrane proximal YxxØ motif blocks AP2 binding to that motif or that the mutation acts via an allosteric mechanism to block AP2 binding to a downstream YxxØ motif (*i.e.*, Y193 or Y204). To distinguish between these two possibilities, the *in vitro* binding of AP2 to GST molecules bearing 21 amino acid fragments of the CD79a cytoplasmic domain centered on each YxxØ motif was determined (Figure 6A). Consistent with the notion that AP2 binds directly to the YxxØ motif centered on the membrane proximal ITAM tyrosine of CD79a (*i.e.*, Y182), AP2 binding to the GST-21-mer possessing only the membrane proximal YxxØ motif is almost as strong as AP2 binding to the complete CD79a cytoplasmic domain. Moreover, mutation of the YxxØ tyrosine of this 21-mer to either alanine or glutamic acid significantly decreases AP2 binding (down to the level of AP2 binding to the intact CD79b cytoplasmic domain, which fails to mediate endocytosis). In contrast, AP2 binding to the 21-mers centered on the two other CD79a YxxØ motifs was negligible and essentially unaltered by mutation of the tyrosine motif. These results establish that, when removed from the influence of CD79b, the membrane proximal ITAM tyrosine of CD79a is capable of binding AP2 directly and driving receptor endocytosis.

The results presented thus far establish that under the right conditions the YxxØ motifs centered on the membrane proximal ITAM tyrosines of CD79a and CD79b can mediate AP2 binding and receptor internalization. Give the striking similarities between the two motifs (they both have the core sequence of YEGL) it was puzzling that they are active under such different conditions (*i.e.*, the membrane-proximal YxxØ of CD79a is only active in isolation whereas the homologous YxxØ of CD79b is only active in the context of the CD79 heterodimer). To further investigate why this might be the case, the GST-21-mer *in vitro* AP2 binding assay was extended to the two YxxØ motifs of CD79b (Figure 6A). Consistent with the finding that the isolated CD79b cytoplasmic domain fails to bind AP2 or mediate internalization (Figure 3), there is little to no appreciable AP2 binding to either of these constructs. However, when one considers the identity of the core membrane proximal YxxØ motifs between CD79a and CD79b, these results suggest that there are likely *cis*-acting regulatory elements within +/−10 amino acids of the membrane proximal ITAM tyrosine residues of both cytoplasmic domains that control AP2 binding and the endocytic activity of these two YxxØ motifs (Figure 6B).

**Figure 7:**
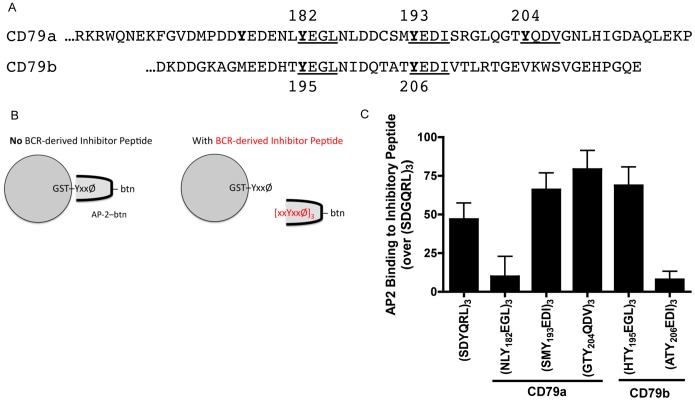
Hierarchical Binding of AP2µ to BCR-derived Minimal YxxØ Motifs. Panel A, Amino acid sequences of CD79a and CD79b cytoplasmic domains. YxxØ putative AP2 binding motifs underlined. Panel B, Diagram of the synthetic peptide–AP2µ binding assay. Panel C, 18 amino acid long peptides of the form [xxYxxØ]_3_ and corresponding to each of the five putative AP2µ binding motifs of CD79 were synthesized and used at a final concentration of 250 µM to block the binding of AP2µ-btn to beads coated with GST-TGN38 (GST–[SDYQRL]_3_). Peptides were also tested across a decreasing range of concentration (Figure S2). Data is presented as the level of BCR-derived inhibitor peptide binding to AP2µ-btn (percent inhibition of binding to bead-associated target) and is the mean of 3 independent experiments ± S.E.M. and data were normalized to the background binding of AP2 to the non-AP2 binding target GST–[SDGQRL]_3_.

To further test the “inherent” AP2 binding properties of the two membrane proximal YxxØ motifs (as well as the other BCR YxxØ motifs), the AP2 binding capacity of synthetic peptides corresponding to each of the five CD79 YxxØ minimal motifs (Figure 7A) was tested. This was accomplished by the approach diagrammed in Figure 7B. First, biotin-labeled AP2µ (AP2µ-btn) was mixed with synthetic peptide corresponding to one of the five BCR-derived YxxØ motifs (the motif was presented in the context of an 18-mer of the form [xxYxxØ]_3_). Next, the mixture of AP2-btn and BCR-derived peptide was added to beads coated with a known AP2 binding domain. If the BCR-derived peptide had bound AP2, it would block binding of AP2 to the beads. The level of AP2µ-btn binding was then determined by SDS-PAGE and blotted with streptavidin-HRP, and percent *inhibition* of AP2µ-btn binding to the beads taken as a read-out of BCR-derived peptide binding to AP2µ-btn.

Using this approach, it became readily apparent that each of the BCR YxxØ motifs has a different inherent capacity to bind AP2 (Figure 7C and Figure S2). Most notable were the peptides corresponding to the two membrane proximal YxxØ motifs of the BCR. The peptide corresponding to the membrane proximal YxxØ of CD79a fails to bind AP2 when presented as a minimal peptide, even though it is active in the isolated CD79a cytoplasmic domain and YxxØ-centered 21-mer (Figures 5 and 6). This suggests that there may be a positive regulatory element within +/−10 amino acids of the core tyrosine that is able to act *in cis* to “activate” the AP2 binding and endocytic activity of this motif (Figure 6B). Conversely, peptide corresponding to the membrane proximal YxxØ motif of CD79b binds AP2 well when presented as a minimal peptide, which is consistent with the activity of this motif in the CD79 heterodimer [Figure 1]. However, the finding that this motif is inactive in the isolated CD79b cytoplasmic domain and YxxØ-centered 21-mer (Figures 3 and 6) suggests that there is a negative regulatory element within +/−10 amino acids of this core tyrosine that is able to act *in cis* to “inactivate” the AP2 binding and endocytic activity of the motif (Figure 6B). Surprisingly, the two downstream YxxØ motifs of CD79a (*i.e.* those centered on Y193 and Y204) also bind AP2 well when presented in isolation, even though they do not appear to contribute to BCR endocytosis when presented either as an isolated CD79a domain or within the context of the CD79 heterodimer (Figures 5 and 1, respectively). In contrast, the single downstream YxxØ motif of CD79b fails to bind AP2 in isolation, consistent with the lack of any data suggesting a role for this motif in BCR internalization. Therefore, there appears to be regulatory elements within 10 amino acids of the membrane proximal ITAM tyrosines of each CD79 subunit, which regulate *in cis* the AP2 binding and endocytic activity of the YxxØ motifs centered on these tyrosines.

### The ITAM-embedded DCSM and QTAT Motifs Control AP2 Binding and Endocytosis

**Figure 8:**
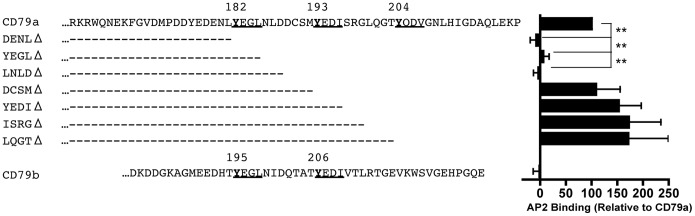
C-terminal Deletions Localize the Motif that Drives AP2 Binding to the Membrane Proximal YxxØ Motif of CD79a. Binding of AP2µ-btn to bead-captured GST bearing the indicated C-terminal deletions of CD79a was determined as in Figure 3. Data is the mean of 3 independent experiments ± S.E.M. and data were normalized to the binding of AP2 to full length CD79a.

To more precisely map the position of the positive regulatory element that is activating AP2 binding and the endocytic activity of the membrane proximal YxxØ of CD79a, the *in vitro* AP2 binding activity of GST fusion proteins bearing C-terminal deletion mutants of the CD79a cytoplasmic domain was determined (Figure 8). Consistent with the lack of AP2 binding to 21-mers centered on CD79a Y193 or Y204 (Figure 6), deletion of any or all amino acids C-terminal to methionine 192 failed to decrease AP2 binding. In contrast, deletion of the DCSM motif (LNLDΔ) located just C-terminal to the Y182-centered YxxØ motif completely ablates AP2 binding, suggesting that DCSM may be the regulatory element that allows AP2 binding to this YxxØ motif.

**Figure 9:**
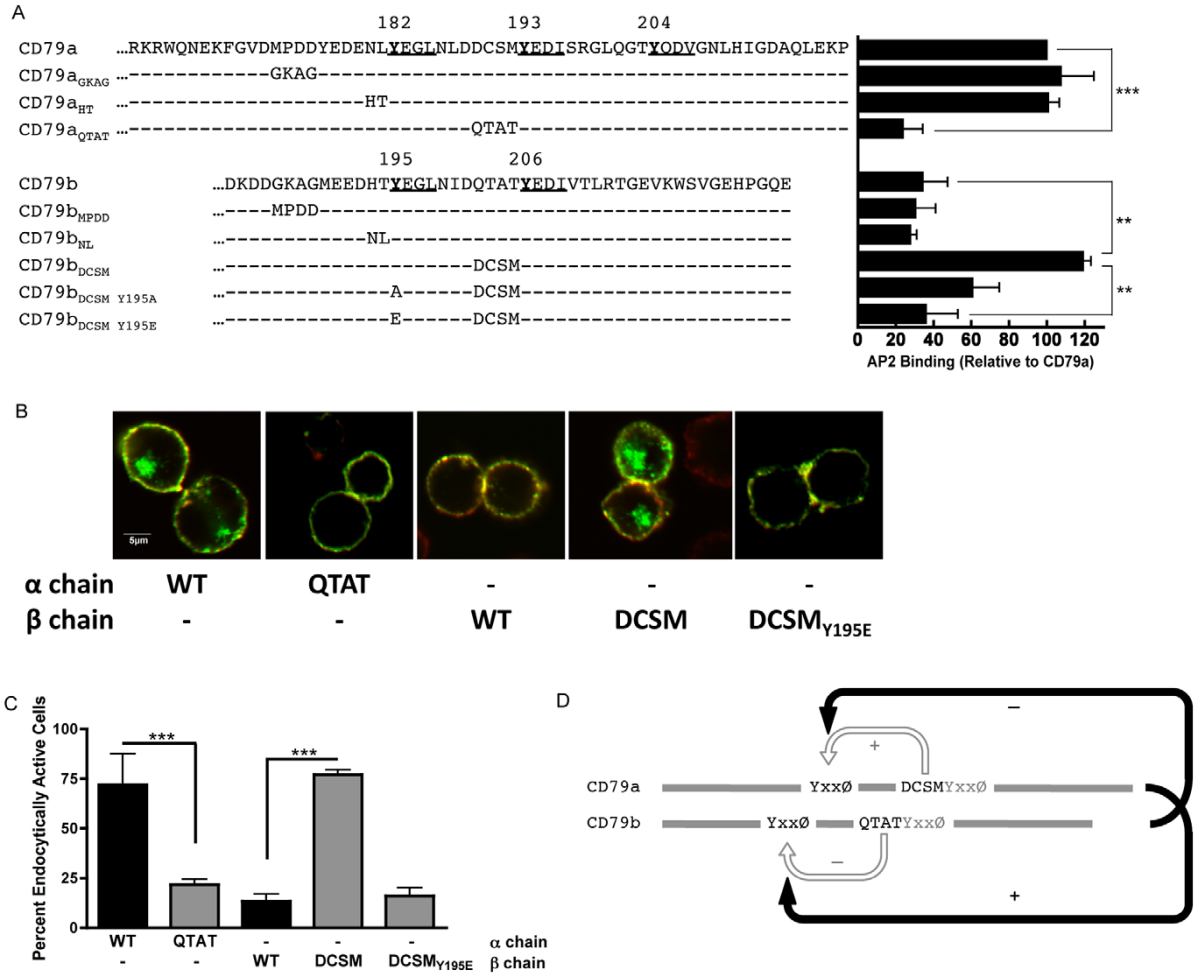
AP2-mediated CD79-driven Endocytosis is Regulated in Cis via ITAM-embedded DCSM and QTAT motifs. Panel A, Binding of AP2-btn to bead-captured GST bearing the indicated CD79a or CD79b cytoplasmic domains with the indicated mutations was determined as in Figure 3. Binding is expressed as a percentage of wild type CD79a binding and represents the mean of 3 independent experiments ± SEM. Statistical comparisons were measured between the non-mutated CD79a or CD79b_DCSM_ cytoplasmic domains and other samples. Panels B and C, Endocytosis of the indicated MHC class II-CD79 fusion proteins was analyzed and quantitated as in Figure 1. Statistical comparisons were made between the reporter protein expressing the wild type CD79a or CD79b cytoplasmic domains and other reporter proteins. Panel D, Diagrammatic representation of *cis* and *trans* regulation of BCR AP2 binding and endocytosis. The open arrows indicate *cis* regulation by the DCSM and QTAT regulatory motifs. The closed arrows represent *trans* regulation of the endocytic activity of each cytoplasmic domain by the presence of the partner cytoplasmic domain.

To determine if the lack of AP2 binding to the LNLDΔ mutant is due to the removal of an activating effect of the DCSM motif or simply due to the deletion of four amino acids, the DCSM and corresponding QTAT region of CD79a and CD79b were swapped and the *in vitro* AP2 binding of the chimeric constructs was tested (Figure 9A). Consistent with the notion that DCSM acts as an activator to “turn-on” the upstream YxxØ motif, replacement of the QTAT in CD79b with DCSM results in a cytoplasmic domain that can now robustly bind AP2. Moreover, mutation of CD79b Y195 to either glutamic acid or alanine abolishes AP2 binding, confirming that the grafted DCSM motif is working *in cis* to activate the AP2 binding properties of the membrane proximal ITAM tyrosine (as opposed to activating a downstream YxxØ motif). When we switch our attention to CD79a, we find that replacement of the DCSM of CD79a with QTAT blocks AP2 binding, suggesting that in the context of an isolated cytoplasmic domain QTAT acts as a negative regulatory element *in cis* to inhibit AP2 binding to upstream YxxØ motifs.

Next, the DCSM/QTAT swap mutants were tested *in situ* by grafting the CD79 cytoplasmic domains onto the MHC class II reporter protein and following internalization of the chimeric molecules (Figures 9B and 9C). Consistent with the *in vitro* AP2 binding results, replacement of the DCSM of CD79a with QTAT results in a block in receptor internalization, whereas replacement of the QTAT of CD79b with DCSM results in a gain of endocytic capacity that is based on the membrane proximal ITAM tyrosine (*i.e.*, CD79b Y195). Therefore, replacement of the QTAT of CD79b with DCSM from CD79a allows the YxxØ motif centered on Y195 of the isolated CD79b domain to act as an AP2 binding site and mediate endocytosis. The results also suggest a potential mechanism for *trans* regulation of AP2 binding that will be discussed below.

## Discussion

BCR internalization is the gateway to targeting BCR-bound antigen to the antigen processing and presentation pathway of the cell and is central to the ability of B cells to recruit T cell help. A recent report on ABC DLBCL established a correlation between increased BCR surface expression (possibly due to decreased BCR internalization) and a higher-than-expected frequency of mutation of the membrane proximal ITAM tyrosine of CD79b [32]. In this report, we extend those studies by demonstrating that the YxxØ endocytosis motif centered on CD79b Y195 is the sole active BCR endocytosis motif, and that mutation of this single residue blocks antigen-driven BCR internalization. Interestingly, another mutation found in a more limited number of ABC DLBCL patients is deletion of a large portion of the CD79a cytoplasmic domain [32]. Given our finding that the presence of the CD79a cytoplasmic domain is necessary to “activate” the endocytic capacity of CD79b, it is tempting to speculate that this subset of ABC DLBCL patients may also have a defect in BCR internalization due to impaired activation of CD79b-mediated BCR endocytosis *in trans*.

The AP2 complex is the most highly characterized endocytic adaptor protein and binding of the YxxØ endocytic motif to the µ subunit of the complex is understood at a molecular level. While previous identification of the CD79b tyrosines present within YxxØ endocytosis motifs as critical for BCR internalization *suggested* a role for AP2 in BCR endocytosis [28], [32], this is the first demonstration that AP2 binds the BCR to mediate cross-linking-induced receptor internalization. Furthermore, as previous studies have established both that tyrosine phosphorylation of YxxØ motifs blocks AP2µ associations [22] and that only a small fraction of CD79-based ITAM tyrosines are targets of phosphorylation during BCR signaling [7], it is not surprising that BCR signaling and endocytosis are mutually exclusive functions of the BCR complex [7]. In fact, Clark and colleagues established that upon antigen binding the minor population of ITAM phosphorylated BCR molecules remains at the cell surface, whereas the major population of cross-linked but non-ITAM phosphorylated BCR molecules is rapidly internalized [7]. Consistent with this notion, we have established that tyrosine phosphorylation of the minimal YxxØ motif corresponding to the membrane proximal ITAM tyrosine of CD79b blocks the ability of this motif to bind AP2 (Figure S3). Thus, tyrosine phosphorylation, which is a transient modification, is at least one molecular mechanism that regulates BCR internalization.

Given the sequence similarity of the membrane proximal ITAM YxxØ motifs of the BCR, it was surprising that CD79a and CD79b possess such dramatically different abilities to interact with AP2 and mediate cross-linking-induced BCR internalization. It appears that this is because multiple mechanisms control BCR endocytosis. In addition to inactivation of AP2 binding by signaling-dependent tyrosine phosphorylation, BCR internalization can be regulated *in cis* by ITAM-embedded DCSM and QTAT motifs and *in trans* by the presence or absence of the partner cytoplasmic domain (Figure 9D). The DCSM and QTAT sequences have been previously implicated in controlling differential signaling by CD79a and CD79b [36]–[38]. In this current study and the related study of Jang et al. [24], replacing the QTAT in CD79b with DCSM results in “activation” of the upstream membrane proximal YxxØ motif to allow AP2 binding to the isolated CD79b cytoplasmic domain and receptor endocytosis. This result implicates DCSM as an activator of upstream YxxØ motif activity. Currently, the mechanism by which DCSM activates AP2 binding is unclear. Analysis of peptides corresponding to the cytoplasmic domains of CD79a and CD79b suggests that they are both relatively unstructured [39]. Nevertheless, *in silico* modeling of the CD79b cytoplasmic domain (Figure S4) predicts a significant tendency toward a β strand structure in the QTAT region of the molecule. Interestingly, modeling of CD79b in which QTAT is replaced by DCSM results in a different predicted structure, with an α helical stretch in the DCSM region of the molecule. This suggests that a DCSM-induced change in the local structure/stability of the cytoplasmic domain may underlie the ability of DCSM to activate the upstream YxxØ motif. On the flip side, molecular modeling of the CD79a cytoplasmic domain failed to reveal a corresponding change in the predicted structure between the DCSM and QTAT swapped cytoplasmic domains. However, the protein structure prediction algorithms may not be sufficiently sensitive to confidently predict any QTAT-induced structural changes. Future studies will test this and other potential mechanisms of *cis* regulation of BCR internalization.

The other mechanism regulating BCR internalization identified in this study is regulation *in trans*. While the precise mechanism of *trans* regulation is currently unclear, consideration of findings from this study along with those from Jang et al. [24] provides some clues. First, Jang et al. found that, similar to this study, the isolated CD79b cytoplasmic domain fails to mediate receptor internalization. However, they paired *two* CD79b cytoplasmic domains (each with its endogenous QTAT inhibitory motif) and found a restoration of BCR internalization, suggesting that the simple presence of a partner cytoplasmic domain may be sufficient to mediate activation *in trans*. Moreover, these results demonstrate that positive regulation *in trans* is dominant over QTAT-based negative regulation *in cis*. This idea is consistent with a preliminary finding that co-expression of CD79a and CD79b cytoplasmic domains wherein both possess either a DCSM or QTAT motif (i.e. CD79a+CD79b_DCSM_ or CD79a_QTAT_+CD79b) results in receptor internalization via the membrane proximal tyrosine of CD79b (data not shown). This confirms and extends the Jang et al. result and means that the DCSM and QTAT motifs appear *not* to be the whole story of regulation of BCR internalization *in trans* (*i.e.*, trans regulation is *not* simply the result of the DCSM CD79a activating the membrane proximal YxxØ in CD79b, while the QTAT of CD79b inhibits the membrane proximal YxxØ in CD79a).

Another clue to the mechanism of *trans* regulation comes from the findings on the endocytic activity of CD79a in which the endogenous DCSM is replaced by QTAT. Here, we report that expression of CD79a_QTAT_ with a very short partner CD79b cytoplasmic domain (3 amino acids, RHR, Table S1) results in a receptor that is endocytically *in*active (due to the impact of the QTAT “inhibitory” motif). However, Jang et al. expressed the CD79a_QTAT_ cytoplasmic domain paired with a slightly longer partner CD79b cytoplasmic domain (∼14 amino acids longer, ending right before the membrane proximal ITAM tyrosine of CD79b, Table S1), resulting in an endocytically *active* receptor. Thus, the presence of a relatively short ∼17 amino acid long fragment of the CD79b cytoplasmic domain devoid of any YxxØ motifs or a QTAT regulatory domain is sufficient to act *in trans* and overcome the inhibitory effect of having grafted the QTAT motif into CD79a. Thus, the mechanism of *trans* regulation of BCR signaling appears to be completely distinct from that of *cis* regulation and likely involves crosstalk between the two cytoplasmic domains (Figure 9D). This general idea is not completely new, as it has been suggested that there is crosstalk between the two cytoplasmic domains of the BCR during signaling [29]. However, this is the first time *trans* regulation has been shown to control receptor internalization.

In summary, we have identified the BCR endocytosis motif, the endocytic adaptor used by the receptor and several mechanisms by which BCR internalization is regulated. In addition to blockade of AP2 binding via tyrosine phosphorylation, the ITAM embedded DCSM and QTAT motifs previously implicated in controlling BCR signaling [36]–[38] act *in cis* to control AP2 binding motif activity. Regulation of BCR internalization also occurs *in trans* via the presence or absence of the partner CD79 cytoplasmic domain. These findings suggest that like BCR signaling, BCR internalization is a highly regulated process that is precisely tuned to give the appropriate response to different degrees of receptor cross-linking. Future studies will provide greater insight into the molecular mechanisms behind this process.

## Supporting Information

Figure S1 **Flow Cytometric Analysis of Reporter Construct Internalization.** Panel A, Amino acid sequences of the cytoplasmic domains of CD79a and CD79b. YxxØ putative AP2 binding motifs underlined. Panel B, Flow cytometric analysis of the endocytosis of the indicated MHC class II-CD79 chimeric protein. Data is the mean of 3 independent experiments (except for the CD79b Y206E sample, which is the mean of 2 independent experiments) ± S.E.M. Statistical comparisons were made between the construct with both CD79 cytoplasmic domains and cells expressing other constructs.
(TIF)

Figure S2 **Titration of BCR-derived Inhibitor Peptides in AP2 Binding Assay.** 18 amino acid long peptides of the form [xxYxxØ]_3_ and corresponding to each of the three AP2µ binding motifs of CD79 (Figure 7) were used across a range of concentrations up to 250 µM to block the binding of AP2µ-btn to beads coated with GST-TGN38 (GST–[SDYQRL]_3_). Data is the mean of 3 independent experiments ± S.E.M. and was normalized to the background binding of AP2 to the non-AP2 binding target GST–[SDGQRL]_3_.
(TIF)

Figure S3 **Tyrosine Phosphorylation Blocks AP2 Binding to BCR-derived Peptides.** 18 amino acid long peptides of the form [xxYxxØ]_3_ and corresponding to the CD79 YxxØ motifs centered on CD79b Y195 were synthesized with tyrosine (Y), phosphotyrosine (pY), glutamic acid (E) or valine (V) in the “Y” position of the YxxØ motif. All were tested for AP2µ binding at 250 µM as in Figure 7. Data is the mean of 3 independent experiments ± S.E.M. and was normalized to the background binding of AP2 to the non-AP2 binding target GST–[SDGQRL]_3_.
(TIF)

Figure S4 **Predicted Structure of CD79 Cytoplasmic Domains.** The amino acid sequences of the cytoplasmic domains of CD79a and CD79b as well as CD79b DCSM (CD79b with QTAT replaced by DCSM) and CD79a QTAT (CD79a with DCSM replaced by QTAT) were uploaded to the Phyre^2^– Protein Homology/analogY Recognition Engine V 2.0 (http://www.sbg.bio.ic.ac.uk/phyre2/html/page.cgi?id=index) and analyzed under the “intensive” modeling mode [40]. Shown is the “Secondary structure prediction” for each of the four sequences. Replacing the DCSM of CD79a with QTAT from CD79b has very little impact on the predicted structure of the cytoplasmic domain, as both are predicted to have a strong α-helical tendency. However, replacing the QTAT of CD79b with DCSM from CD79a induces the formation of a short run of predicted α-helical structure immediately downstream of the membrane-proximal YxxØ. Since this region of wild type CD79b is predicted to have a strong β-strand tendency, these results suggest that DCSM may have an effect on domain structure that is favorable for AP2 binding. A similar predicted secondary structure, with an “induced” short α-helical run in CD79b DCSM was observed when the same sequences were analyzed with the I-TASSER structure prediction algorithm (http://zhanglab.ccmb.med.umich.edu/I-TASSER/) [not shown] [41].
(TIF)

Table S1 **Comparison of Cytoplasmic Domains Used to Test BCR Endocytosis.**
(TIF)
