# Supplementary figures and images for: Cis and Trans Regulatory Mechanisms Control AP2-Mediated B Cell Receptor Endocytosis via Select Tyrosine-Based Motifs
Source: PLoS One. 2013 Jan 23;8(1):e54938. doi: 10.1371/journal.pone.0054938 (PMC3553015; doi:10.1371/journal.pone.0054938)

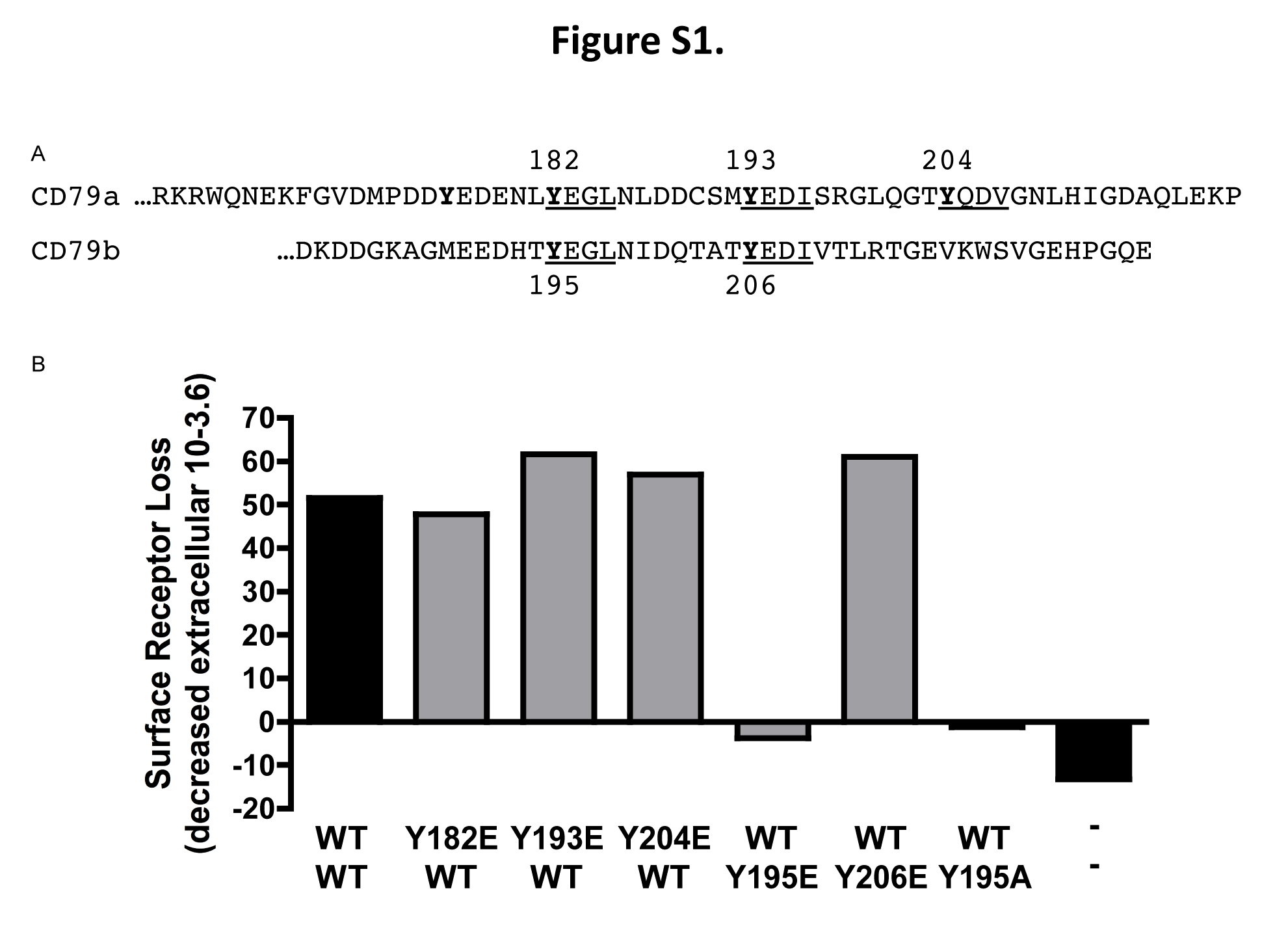

Supplement: Figure S1 — Flow Cytometric Analysis of Reporter Construct Internalization. Panel A, Amino acid sequences of the cytoplasmic domains of CD79a and CD79b. YxxØ putative AP2 binding motifs underlined. Panel B, Flow cytometric analysis of the endocytosis of the indicated MHC class II-CD79 chimeric protein. Data is the mean of 3 independent experiments (except for the CD79b Y206E sample, which is the mean of 2 independent experiments) ± S.E.M. Statistical comparisons were made between the construct with both CD79 cytoplasmic domains and cells expressing other constructs. (TIF) [file pone.0054938.s001.tif]

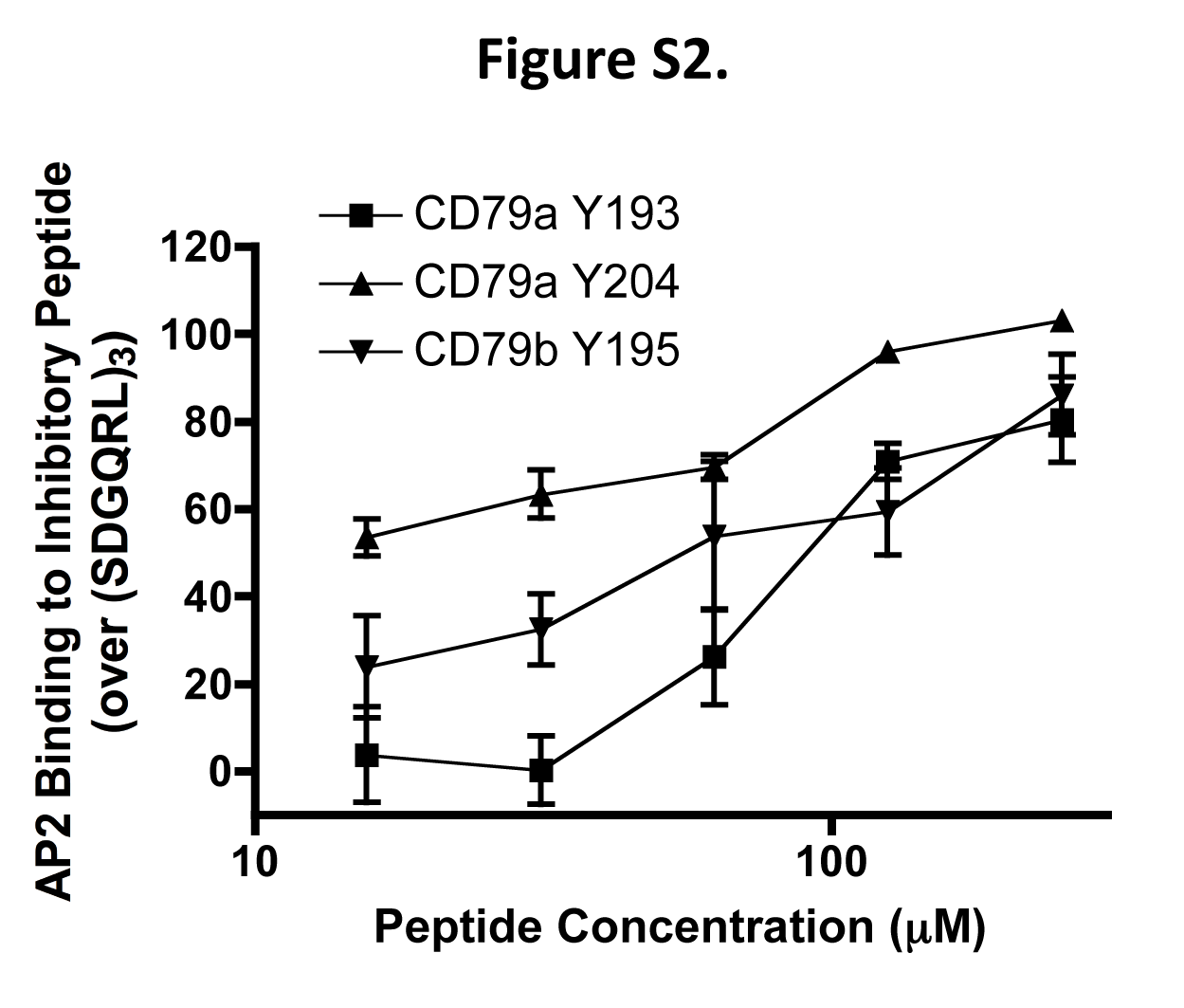

Supplement: Figure S2 — Titration of BCR-derived Inhibitor Peptides in AP2 Binding Assay. 18 amino acid long peptides of the form [xxYxxØ]3 and corresponding to each of the three AP2µ binding motifs of CD79 (Figure 7) were used across a range of concentrations up to 250 µM to block the binding of AP2µ-btn to beads coated with GST-TGN38 (GST–[SDYQRL]3). Data is the mean of 3 independent experiments ± S.E.M. and was normalized to the background binding of AP2 to the non-AP2 binding target GST–[SDGQRL]3. (TIF) [file pone.0054938.s002.tif]

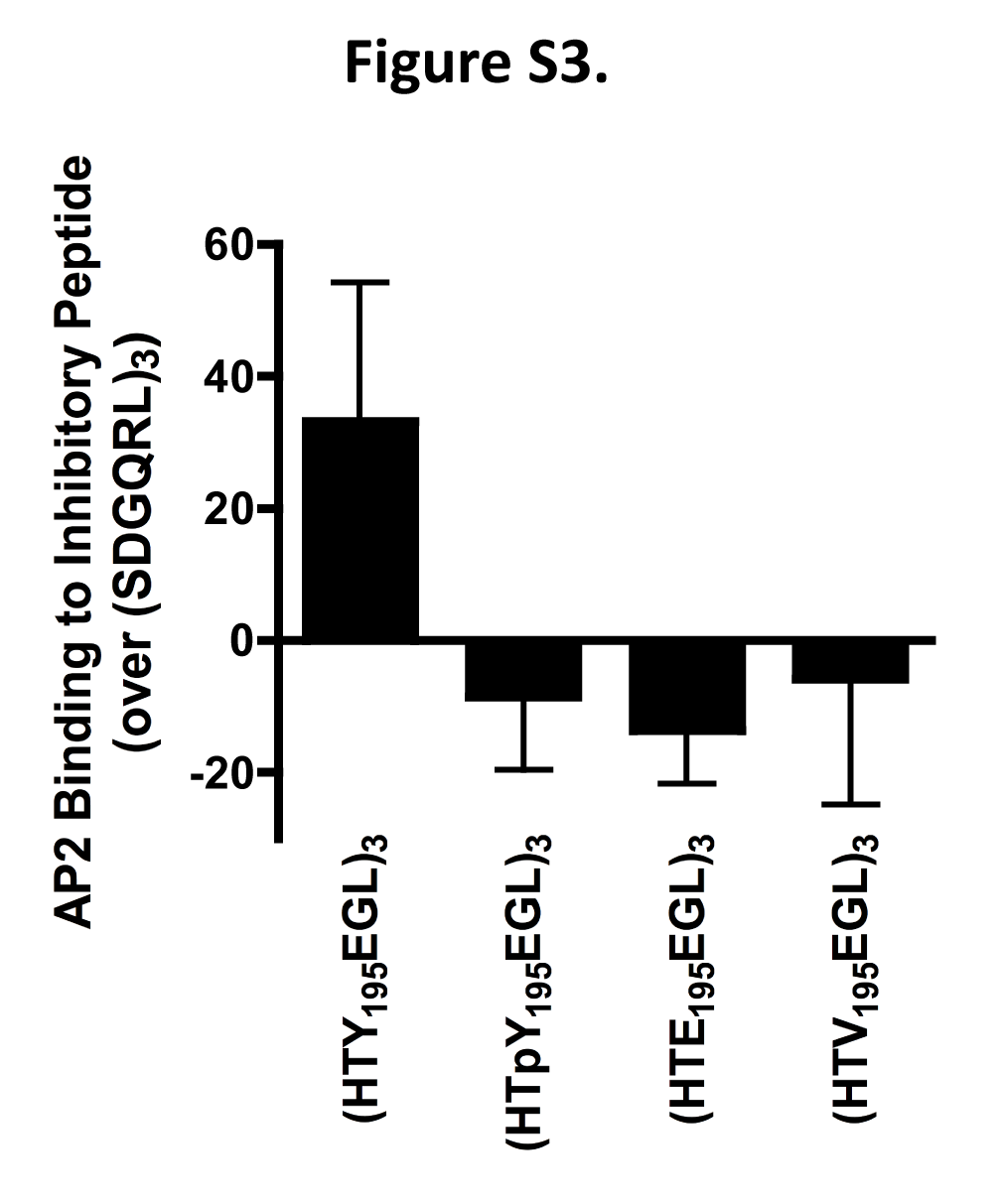

Supplement: Figure S3 — Tyrosine Phosphorylation Blocks AP2 Binding to BCR-derived Peptides. 18 amino acid long peptides of the form [xxYxxØ]3 and corresponding to the CD79 YxxØ motifs centered on CD79b Y195 were synthesized with tyrosine (Y), phosphotyrosine (pY), glutamic acid (E) or valine (V) in the “Y” position of the YxxØ motif. All were tested for AP2µ binding at 250 µM as in Figure 7. Data is the mean of 3 independent experiments ± S.E.M. and was normalized to the background binding of AP2 to the non-AP2 binding target GST–[SDGQRL]3. (TIF) [file pone.0054938.s003.tif]

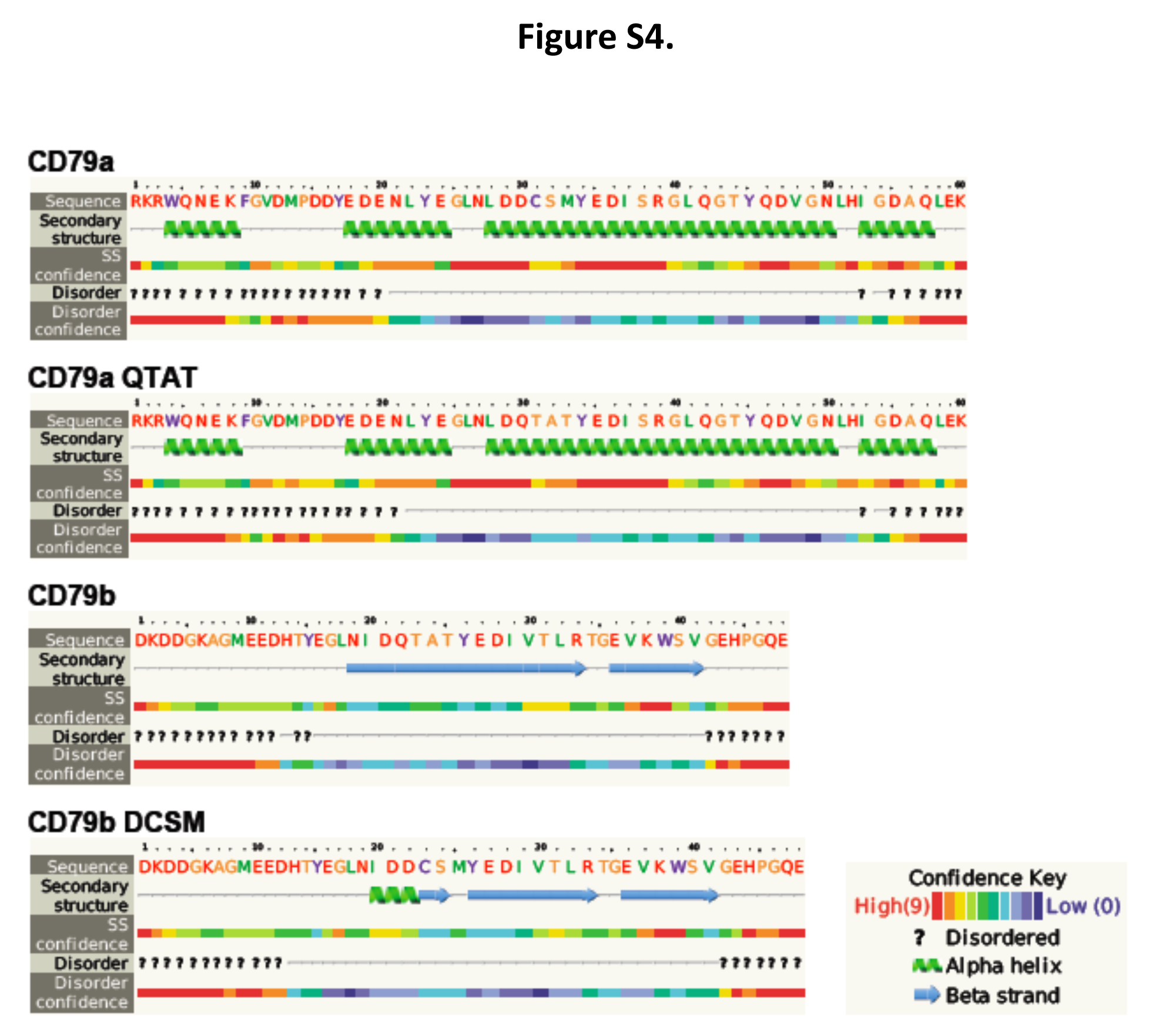

Supplement: Figure S4 — Predicted Structure of CD79 Cytoplasmic Domains. The amino acid sequences of the cytoplasmic domains of CD79a and CD79b as well as CD79b DCSM (CD79b with QTAT replaced by DCSM) and CD79a QTAT (CD79a with DCSM replaced by QTAT) were uploaded to the Phyre2– Protein Homology/analogY Recognition Engine V 2.0 (http://www.sbg.bio.ic.ac.uk/phyre2/html/page.cgi?id=index) and analyzed under the “intensive” modeling mode [40]. Shown is the “Secondary structure prediction” for each of the four sequences. Replacing the DCSM of CD79a with QTAT from CD79b has very little impact on the predicted structure of the cytoplasmic domain, as both are predicted to have a strong α-helical tendency. However, replacing the QTAT of CD79b with DCSM from CD79a induces the formation of a short run of predicted α-helical structure immediately downstream of the membrane-proximal YxxØ. Since this region of wild type CD79b is predicted to have a strong β-strand tendency, these results suggest that DCSM may have an effect on domain structure that is favorable for AP2 binding. A similar predicted secondary structure, with an “induced” short α-helical run in CD79b DCSM was observed when the same sequences were analyzed with the I-TASSER structure prediction algorithm (http://zhanglab.ccmb.med.umich.edu/I-TASSER/) [not shown] [41]. (TIF) [file pone.0054938.s004.tif]

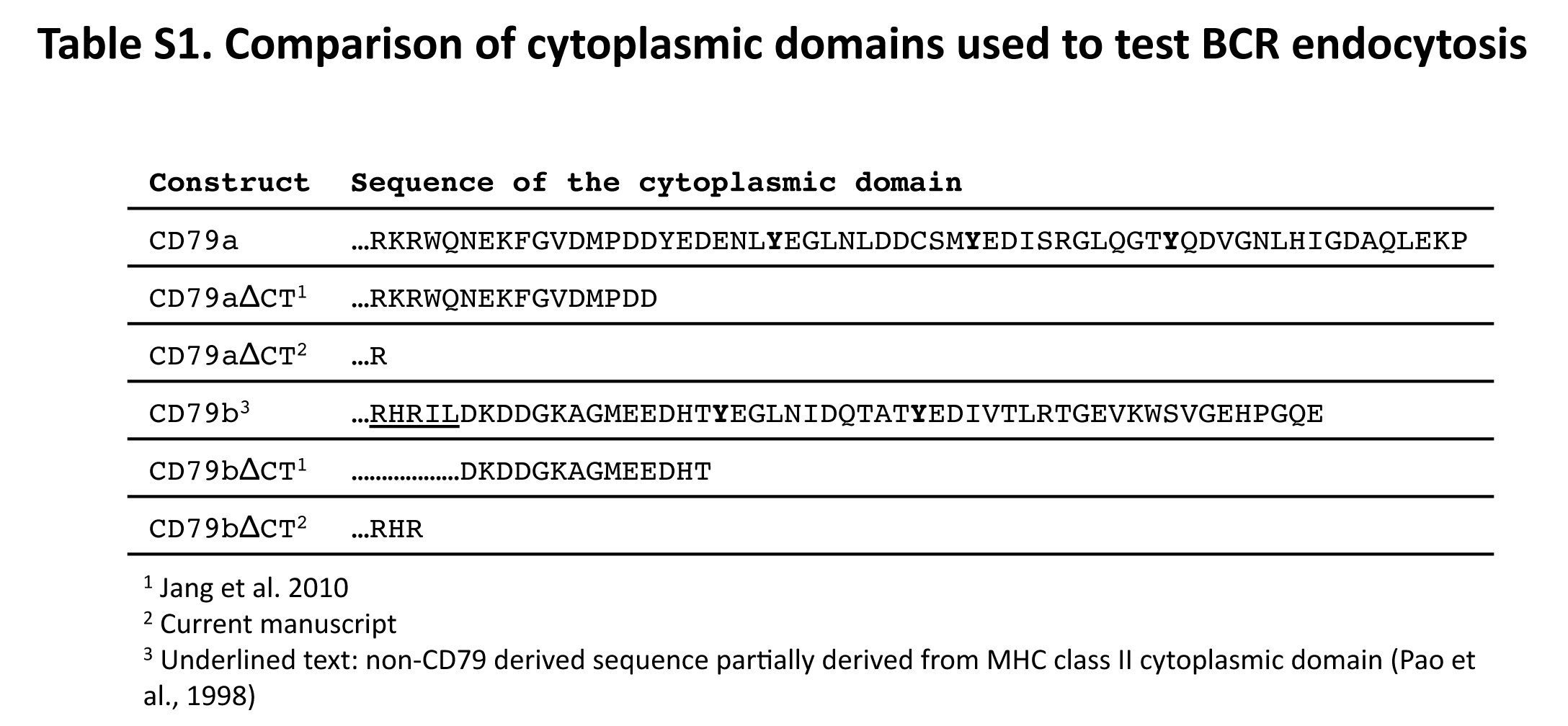

Supplement: Table S1 — Comparison of Cytoplasmic Domains Used to Test BCR Endocytosis. (TIF) [file pone.0054938.s005.tif]
